# 3D-Printing of Artificial Aortic Heart Valve Using UV-Cured Silicone: Design and Performance Analysis

**DOI:** 10.3390/bioengineering12010094

**Published:** 2025-01-20

**Authors:** Atila Ertas, Erik Farley-Talamantes, Olkan Cuvalci, Ozhan Gecgel

**Affiliations:** 1 Department of Mechanical Engineering, Texas Tech University, Lubbock, TX 79409, USA; erik.farley@ttu.edu (E.F.-T.); ozhan.gecgel@ttu.edu (O.G.); 2 Department of Mechanical Engineering, Karadeniz Technical University, 61080 Trabzon, Türkiye; ocuvalci@ktu.edu.tr

**Keywords:** 3D printing, polymeric heart valve production, silicone 3D printing machine design, aortic heart valve design

## Abstract

The advancement of medical 3D printing technology includes several enhancements, such as decreasing the length of surgical procedures and minimizing anesthesia exposure, improving preoperative planning, creating personalized replicas of tissues and bones specific to individual patients, bioprinting, and providing alternatives to human organ transplants. The range of materials accessible for 3D printing within the healthcare industry is significantly narrower when compared with conventional manufacturing techniques. Liquid silicone rubber (LSR) is characterized by its remarkable stability, outstanding biocompatibility, and significant flexibility, thus presenting substantial opportunities for manufacturers of medical devices who are engaged in 3D printing. The main objective of this study is to develop, refine, and assess a 3D printer that can employ UV-cured silicone for the fabrication of aortic heart valves. Additionally, the research aims to produce a 3D-printed silicone aortic heart valve and evaluate the feasibility of the final product. A two-level ANOVA experimental design was utilized to investigate the impacts of print speed, nozzle temperature, and layer height on the print quality of the aortic heart valve. The findings demonstrated that the 3D-printed heart valve’s UV-cured silicone functioned efficiently, achieving the target flow rates of 5 L/min and 7 L/min. Two distinct leaflet thicknesses (LT) of the heart valve, namely 0.8 mm and 1.6 mm, were also analyzed to simulate calcium deposition on the leaflets.

## 1. Introduction

Recent advancements in 3D printing technologies are revolutionizing the creation of economical, lightweight, and durable medical components in healthcare [1,2] These cutting-edge technologies enable the production of components and products that are more cost-effective, safer, lighter, and more robust. A critical aspect of this capability lies in the material’s compatibility with existing 3D printing technologies, as well as its ability to adapt the manufacturing process to accommodate the material [3].

Over the past two decades, 3D printing has made remarkable progress. Additive manufacturing has facilitated significant advancements in the field of complex medical engineering, allowing for the development of artificial organs, tissues, and various other bodily components [4,5,6]. The evolution of medical 3D printing technology encompasses various improvements, such as reducing surgical durations and limiting anesthesia exposure, enhancing preoperative planning, producing customized replicas of tissues and bones tailored to individual patients, bioprinting, and offering alternatives to human organ transplants. The integration of 3D printing with handheld ultrasonography, CT imaging, and X-ray technology has transformed the landscape of medical practices [7].

Heart valves can be classified into three main categories: mechanical valves, which are constructed from synthetic materials such as carbon, titanium, or polymers; tissue-engineered heart valves (TEHV), which utilize animal or human tissues that have been processed to minimize the risk of rejection by the body and are also referred to as bioprosthetic valves; and polymeric heart valves (PHV), which are composed of biocompatible and biostable polymer materials. It is important to highlight that both TEHV and PHV can be manufactured using 3D printing technology.

Three primary domains in which 3D printing has notably enhanced healthcare are outlined below [7]:

Implants: The field of implant manufacturing has undergone a significant transformation due to the advent of 3D printing technology, enabling the creation of devices tailored to the specific needs of individual patients. This innovative approach allows for precise production and meticulous design of implants used in dental, spinal, and hip applications.
Prostheses: The subject of prosthetics is an area in which 3D printing has profoundly influenced the healthcare sector. Through 3D printing, prosthetic limbs can be customized rapidly and cost-effectively to address the unique requirements of each individual.
Surgical Tools: Surgeons may employ tailored surgical instruments produced through 3D printing to align with a patient’s specific anatomy or treatment requirements. This degree of customization minimizes the risk of errors, accelerates the recovery process for patients, and reduces tissue trauma.

Recent studies indicate that 3D printing is increasingly recognized for its potential in the production of heart valves [8,9,10]. The capability of 3D printing technology to create complex, customized structures could significantly revolutionize the heart valve manufacturing process [10]. Ventricular heart disease, a significant contemporary health issue, impacts 3.1% of the adult population [11]. The predominant treatment approach involves replacing the damaged valve with an artificial one. However, the challenge of designing a heart valve is considerable as the aortic valve experiences between 30 and 40 million cycles annually and pumps approximately 7600 L of blood each day [12].

The present emphasis on advancing 3D-printed heart valves aims to enhance their functional performance, customization, and biocompatibility. Investigators are exploring innovative materials and manufacturing techniques to accurately mimic the complex structure and functionality of human heart valves [8]. Recent breakthroughs are set to transform cardiac valve replacement procedures, offering long-term, tailored care for individuals suffering from heart valve disorders [13].

The emphasis on patient-specific design marks a significant advancement in the development of 3D-printed heart valves. Researchers can leverage medical imaging modalities, such as CT scans and MRIs, to create tailored models of an individual’s heart, enabling the production of customized heart valves that fit their unique anatomical structure. This methodology aims to enhance the overall effectiveness and compatibility of the implant [14]. The existing literature indicates that 3D printing technology is still in the early stages with regard to biological and technological requirements. A primary concern remains to be the assurance of biocompatibility of the heart valves produced through 3D printing.

**Figure 1:**
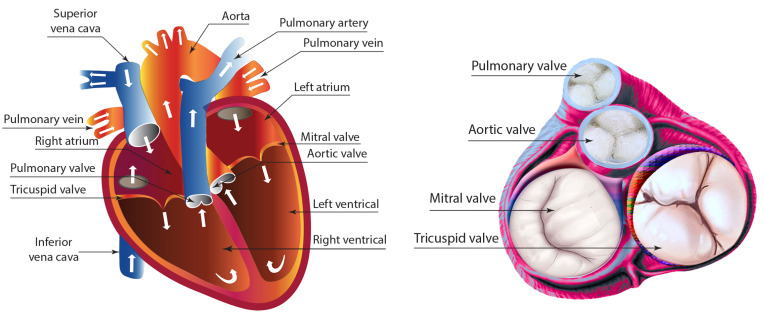
Illustration of four primary heart valves.

The valves of the heart serve a crucial role in regulating blood circulation. As illustrated in Figure 1, the heart consists of four primary valves: the mitral valve (also known as the bicuspid valve) and the tricuspid valve, which are classified as atrioventricular valves. Additionally, the aortic valve located in the aorta and the pulmonary valve situated in the pulmonary trunk represent the two semilunar valves found within the arteries.

When heart valves operate correctly, blood flows through the heart in one direction. The valves open to facilitate the movement of blood from one chamber to another and close to inhibit any backflow to the previous chamber, ensuring no leakage occurs. In cases where the leakage is minimal, individuals often do not exhibit any symptoms. However, if the condition deteriorates, the heart is required to exert more effort to circulate blood throughout the body. This increased strain over time can lead to a weakening of the heart muscle, potentially resulting in significant health complications.

**Figure 2:**
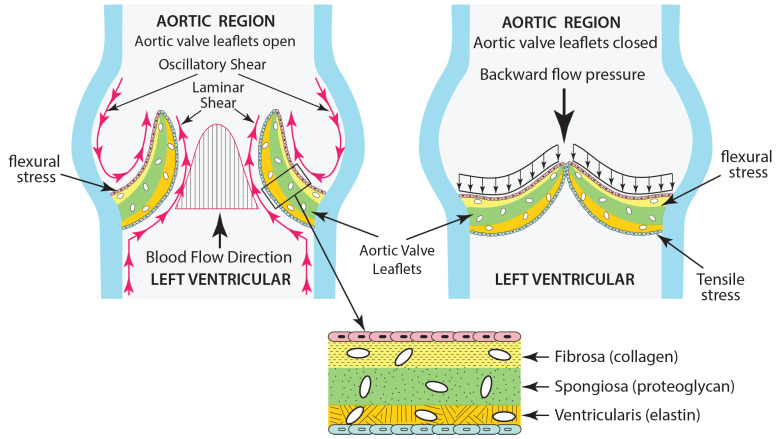
Stresses on three distinct layers of structures of aortic heart valve leaflets.

Aortic valves exhibit a complex architecture comprising three distinct layers of structures (refer to Figure 2), which are designed to efficiently accommodate the dynamic mechanical stresses generated throughout the cardiac cycle. The three layers are detailed by Amindari et al. [15]:

1. The fibrosa layer, located at the outflow surface, consists of collagen fibers, which provide structural strength.
2. The ventricularis layer located on the inflow surface is composed of elastin, which allows the valve to expand and contract during the cardiac cycle.
3. The spongiosa layer is situated centrally and consists of loose connective tissue, which facilitates the relative movement of the adjacent layers.

The aortic valves, which facilitate the passage of blood from the left ventricle to the aorta, consist of resilient, thin tissue structures referred to as leaflets. These leaflets operate by opening and closing according to the heart’s pumping action. This action generates dynamic loading, which will result in the following [16,17]:

- Shear stresses resulting from blood flow occur when the valve is in the systolic (open) position.
- Flexural stress induced by transvascular pressure during the processes of opening and closing.
- Tensile stresses arise when the valve is in a closed position during diastole.

### Recent Advancements in 3D Printed Aortic Heart Valves Utilizing Various Materials

Polymeric heart valves [18,19], composed of flexible, synthetic materials, were among the earliest types of artificial heart valve replacements.

While many challenges remain to overcome before bioengineered heart valves can be produced, scientific progress has been accomplished thus far.

**Figure 3:**
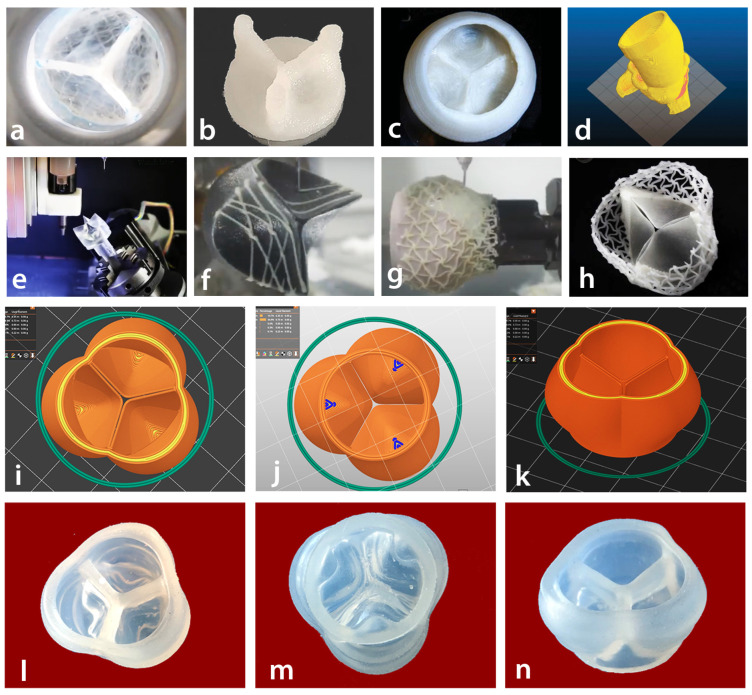
Recent advancements in 3D printing of an aortic heart valve using various materials.

Figure 3 illustrates recent advancements in 3D-printed aortic heart valves utilizing different materials.

The team at the Harry Perkins Institute of Medical Research has created a heart valve by using particular biopolymers, which are natural polymers derived from the cells of living organisms and produced via 3D printing technology (see Figure 3a). Researchers at the institute have found that bioinspired valves demonstrate superior performance and durability compared to existing heart valves [20].

The CHU Sainte-Justine Research Centre has created composite inks that incorporate both synthetic and natural polymers, such as PVA, Gel, and κ-carrageenan (CG). The findings indicated that the developed inks possessed suitable rheological characteristics for extrusion-based 3D printing, enabling precise printing of human-scale heart valves [21]. Various sizes and types of heart valves are successfully printed with high fidelity in the air (see Figure 3b).

Researchers at Carnegie Mellon University (CMU) have developed functional heart components through an advanced 3D bioprinting method [22]. The research results demonstrate that the use of freeform reversible embedding of suspended hydrogels (FRESH) in 3D bioprinting enables the creation of hearts that precisely mirror the anatomical characteristics of individual patients, as confirmed through micro-computed tomography analysis (see Figure 3c).

Researchers at the University of Minnesota have made notable advancements in Transcatheter Aortic Valve Replacement (TAVR) by utilizing 3D printing technology to create an accurate model of a patient’s affected valve and the adjacent sections of the aorta. The research team employed CT imaging of the patient’s heart to accurately reproduce the specific anatomy of the aortic root area (see Figure 3d). The next step involves 3D printing using specialized silicone-based inks that replicate the texture of actual heart tissue [23].

Using multiple production phases, artificial heart valves are manufactured by Visco Tec through silicone additive manufacturing [24]. The process starts by spraying soft silicone onto a three-pointed crown, which forms the valve’s thin leaflets. Subsequently, several layers are employed to form the valve leaflets (refer to Figure 3e). Following this, a 3D printer applies a pliable silicone paste to produce designated patterns of fine threads on the surface of the leaflets, thereby enhancing their structural integrity (see Figure 3f,g). Figure 3h shows the finished product of the aortic heart valve.

In this study, UV-cured silicone was employed in the production of a heart valve. The curing of silicone occurs through exposure to UV light, with the cross-linking process initiated by a photochemical reaction instead of heat. Figure 3i–k illustrates the digital model of the aortic heart valve (refer to Section 2.3 of this paper). In contrast, Figure 3l–n present different perspectives of the manufactured heart valve. Figure 3l shows the top view, Figure 3m displays the bottom view, and Figure 3n represents the side view.

The multiphase production process is closely aligned with this paper’s 3D printing method for heart valve production. Both methodologies employ silicone as a fundamental material. While the multiphase production process utilizes photo-curable silicone, this research focuses on UV-cured silicone. A notable advantage of this research is the adoption of a single production process, in contrast to a multiphase production approach. The 3D printing method illustrated in Figure 3d shares similarities with the approach outlined in this study; however, it employs specialized silicone-based inks rather than UV-cured silicone. It is important to highlight that material suppliers strive to innovate new silicone elastomers to reduce both time and costs in manufacturing, potentially enabling businesses to eliminate the need to post-cure their silicone components [25].

The range of materials available for 3D printing in the healthcare sector is notably limited when compared to traditional manufacturing methods. Although researchers are continuously striving to expand the selection of materials, certain medical-grade substances may not be compatible with 3D printing processes. Ensuring consistent quality in 3D-printed medical devices presents a significant challenge as fluctuations in print quality or material properties can adversely affect the reliability and safety of medical equipment [7].

Elastomers are crucial in biomedical implants due to their flexibility, which mimics soft tissues. In 2021, the worldwide usage of medical elastomers for biomedical implants, including those produced through 3D printing, reached 2900 kilotons. Forecasts suggest a steady rise, with a compound annual growth rate of 6.5% anticipated from 2022 to 2027 [26]. Elastomers can be classified into two main categories based on their cross-linking mechanisms. Physically cross-linked elastomers are referred to as thermoplastic elastomers (TPEs). TPEs are plastics characterized by their ability to stretch and subsequently revert to their original form. Thermoplastic Polycaprolactones (PCL) are extensively used in the medical field due to their biocompatibility. They are particularly useful in 3D printing pericardial scaffolds for treating dilated cardiomyopathy and reducing the risk of congestive heart failure.

Chemically cross-linked elastomers, called thermoset elastomers, maintain their shape after curing and are insoluble. They find applications in tires, drive belts, gaskets, biomedical devices, biological components, composite materials, circuit boards, and electrical insulators. Silicone is a type of thermoset material that cannot be reshaped after its initial formation due to its permanent cross-linking [27]. There are three primary types of silicone elastomers: Liquid silicone rubber (LSR), which is perfect for 3D printing because of its low viscosity; Fluorosilicone rubber (FSR), which is noted for its high resistance to temperatures, fuels, oils, and solvents; and Heat-cured rubber (HCR), which hardens when heated, offering superior thermal stability and mechanical strength.

In 1950, silicone was first used for urethral implantation. Subsequently, it was applied in the development of interphalangeal joint replacement prostheses and shunts. The silicone breast implant became broadly accessible in 1962, with technological advancements continuing into the 1990s [28].

This study aims to design and develop a 3D silicone printer capable of fabricating components with intricate geometries, such as heart valves, which closely mimic the structure and functionality of natural human heart valves. The research will explore the specific type of silicone utilized in this investigation to determine if it produces optimal outcomes, given that different silicone varieties exhibit unique material characteristics. The study will focus on three primary parameters, print speed, nozzle temperature, and layer height, all while maintaining a constant nozzle size of 0.4 mm throughout the experiment to ensure a high degree of precision. A two-level experimental design will be implemented to evaluate the relative impacts of speed, nozzle temperature, and layer height on the print quality of the aortic heart valve. Additionally, two distinct leaflet thicknesses (0.8 mm and 1.6 mm) will be analyzed to simulate calcium accumulation. The functionality of the heart valve will be assessed to determine if it can achieve the target flow rates of 5 L/min and 7 L/min.

## 2. Materials and Methods

### 2.1. Silicone Material

Silicone, a recent addition to 3D printing, has a versatile chemical structure that can be customized for various industrial uses. These include wearables, medical devices, robotic grippers, kitchen tools, thermal and electrical insulation, and sealing solutions [29,30]. A new method now allows silicone to be used as a 3D printing material. Liquid silicone rubber (LSR), known for its stability and resistance to extreme temperatures and conditions, offers potential for manufacturers in 3D printing.

The silicone used for this study is RTV 800-245 UV Cure silicone, provided by NOVAGARD (Cleveland, OH, USA). This silicone is cured using UV light, with cross-linking initiated by a photochemical process instead of heat. RTV 800-245 was selected due to its UV-only cure chemistry, a cure time of 3–5 s, and a viscosity of 4.4 (Pa-sec), as detailed in Table 1. The rapid curing time and low viscosity made it ideal for use in the silicone 3D printer design. Silicone is used for heart valve production in this study due to its tissue compliance and biocompatibility. While known for its biocompatibility, these silicone valves will initially serve as models to simulate bioprosthetic valves. Future modifications may enable their use as implantable synthetic heart valves [24].

Three-dimensional printing technology allows for the production of heart valve models that replicate the precise shape and texture of a patient’s valve. These models are instrumental for physicians in evaluating the optimal size and placement of the valve during surgical interventions.

### 2.2. Tensile Testing of Silicone

The flexibility and mechanical properties of silicone materials are essential for their application to print heart valves. Nevertheless, as indicated in Table 1, the tensile strength of these silicone materials is relatively low, with values ranging from 0.207 to 0.621 MPa. This test aimed to investigate whether changes in UV pre-curing and post-curing durations would influence the mechanical performance of the silicone utilized in this research.

**Figure 4:**
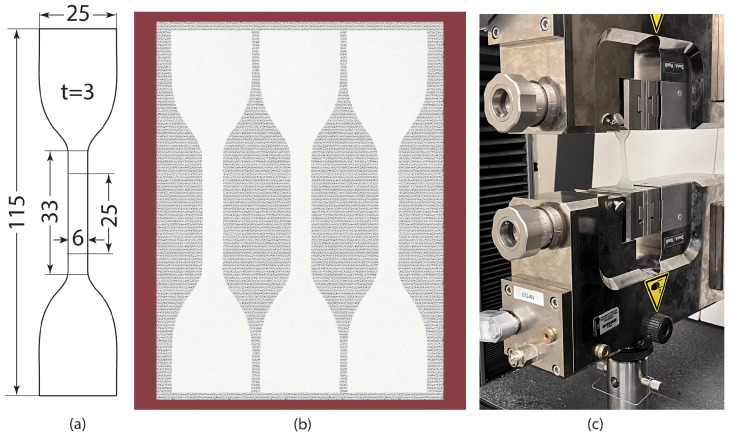
(**a**) Dimensions of the tensile test specimen according to ASTM D412. (**b**) Mold to cast silicone material into a dumbbell shape for a tensile test. (**c**) Sample in between the tensile machine grips (dimensions are not to scale and all the dimensions are in mm).

Tensile testing, as illustrated in Figure 4c, was employed to ascertain the stress–strain region. The specimens utilized in this study adhere to ASTM D412 standards, featuring an overall length of 115 mm, a gauge length of 25 mm, and a thickness of 3 mm as depicted in Figure 4a. A strain rate of 500 mm/min was used for the testing. The silicone was poured into a 3 mm mold (shown in Figure 4b) and tilted to eliminate any trapped air bubbles. To achieve a smooth surface finish, surplus silicone was carefully removed with an acrylic roller.

### 2.3. Geometry Generation for the Heart Valve and the Aorta

The geometry of the aorta was represented as a tri-petal configuration, as detailed by Gulbulak et al. (2020). The geometries of the polymeric heart valves (PHV) were characterized by a combination of three fundamental curves: the attachment curve, the belly curve, and the free edge. While the belly curve and free edge were created solely as three-dimensional splines, the attachment curve was developed by converting a two-dimensional spline into a three-dimensional spline. A cubic Bézier curve was employed to formulate the two-dimensional spline for the attachment curve [31].

**Figure 5:**
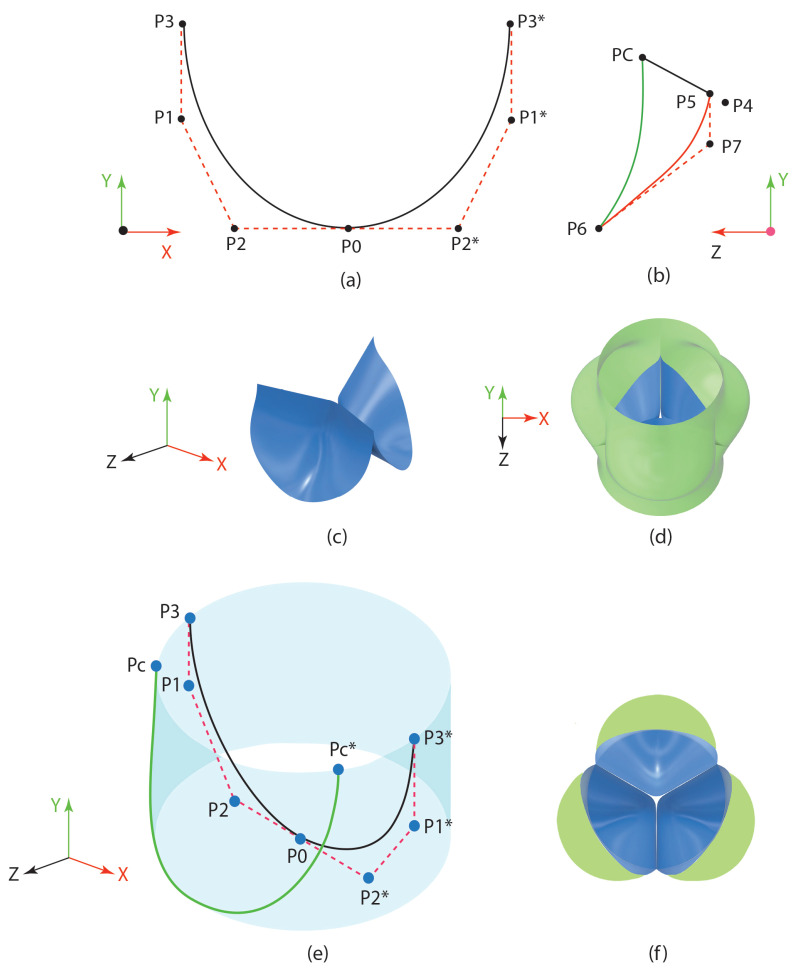
Geometry generation for the heart valve and the aorta (recreated from [31]).

The definition of the 2D spline encompassed four unique points. In Figure 5a, P1 and P2 serve as the control points, while P0 and P3 are designated as the fixed endpoints. Mirrored coordinates are indicated by asterisks. As illustrated in Figure 5a, a 3D curve was generated by wrapping the 2D attachment curve around a cylinder with a diameter of 23 mm [31]. The commissural points of the 3D attachment curve, labeled as PC, along with the center of the valve, denoted as P4, were employed to establish the free edge (refer to Figure 5b). Furthermore, P6 and P5 were identified as the fixed endpoints, while P7 served as the sole control point for defining the belly curve (see Figure 5b). This belly curve acts as a reference for shaping the concave configuration of the valve geometries. The valve leaflets’ surface was designed as boundary patches, incorporating both the attachment and free edge. A rotation of 120° around the origin resulted in three identical surfaces (Figure 5c). The diameters of both the aorta and the valve measured 23 mm. The surface of the aorta was constructed using four circular and three petal-shaped cross-sections (Figure 5d). By adjusting the control points P1, P2, and P7, various geometries of heart valves can be produced.

In Figure 5e, the blue 23 mm cylinder is used as the construction surface. The 2-dimensional attachment curve (in black) is converted into a 3-dimensional curve (in green), and the valve surface is extended to form a complete connection to the aortic surface (Figure 5f). For more information on heart valve and aorta geometry generation, refer to reference: Gulbulak et al. [31,32].

### 2.4. Analysis of Variance (ANOVA)

The analysis of variance (ANOVA) is a widely utilized quantitative technique for global sensitivity analysis, which is particularly effective in assessing the contributions of various factors and their interactions within experimental design [33]. The evaluation of the primary parameters (factors) and their interactions was conducted using orthogonal arrays (OA) [34].

In this research, the L_8_ orthogonal array (OA), which is a two-level model comprising three factors, was employed. This investigation sought to evaluate the relative effects of the most significant parameters—print speed, nozzle temperature, and layer height—on the print quality of the aortic heart valve while keeping the nozzle size of 0.4 mm constant. The identification of these critical parameters was the result of numerous experimental trials. When denoting ‘*k*’ as the number of factors involved in the experiment, a two-level OA with three primary factors results in $2^{k}=2^{3}=8$ data points, as illustrated in Table 2. The primary factors selected for investigation regarding their impact on print quality include print speed (P), nozzle temperature (N), and layer height (L) of the silicone, which are assigned to columns 1, 2, and 4, respectively. The interactions among these three main factors P × N, P × L, and N × L are presented in columns three, five, and six. Column 7 details the accumulation of errors. An ANOVA methodology was utilized by varying the values of the main factors from low (1) to high (2). In Table 2, $Y_{i}$ denotes the observed performance related to print quality.

The primary factors’ lower (1) and upper (2) limits are determined following extensive experimentation. These limits are presented in Table 3.

### 2.5. Direct Observation Method

The most effective and widely accepted method of direct observation was employed to evaluate the quality of the 3D-printed heart valve. The model intended for printing was utilized to assess dimensional accuracy, which formed the foundation for the quality evaluation. To minimize bias, the determination of the print quality of the heart valves involved the input of three different observers. In addition to visual assessment, the second criterion, “stiffness of the heart valve leaflets”, was applied when the print quality was similar and when decision-making proved to be difficult.

The quality of the heart valve print was determined through direct observation [35]. The initial phase involves assessing and confirming the dimensional accuracy of the heart valve while also gaining an overall understanding of its dimensions, including diameter, thickness, and shape. The subsequent phase is orientation, during which the observations are analyzed, findings are evaluated, and future actions are deliberated. A high level of situational awareness and understanding is essential for making an informed decision at this stage. The final phase is decision-making, where all potential outcomes are considered, leading to the formulation of a recommended course of action or response strategy. This process was facilitated through discussions among three observers who collectively evaluated and reached a conclusion regarding the dimensional precision of the eight experimental results ($Y_{i}$) presented in Table 2.

### 2.6. Experimental Setup

**Figure 6:**
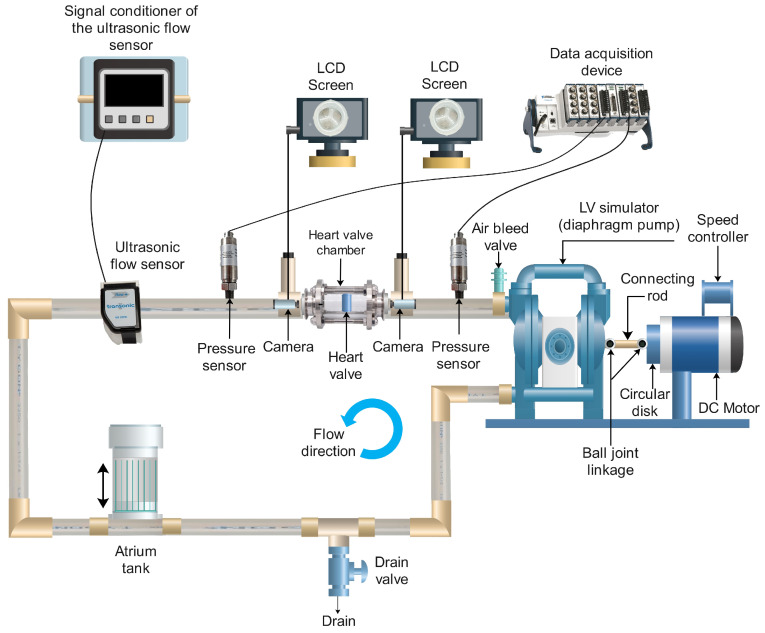
Mock Circulatory Loop experimental setup for blood circulation.

The experimental setup shown in Figure 6 gathers data on cardiac cycles (RPM), pressure readings taken before and after the heart valve was inserted, and flow rate data associated with the thickness of each heart valve leaflet. The combination of water (60%) and glycerin (40%) is commonly employed in Mock Circulatory Loop (MCL) applications because of its previously validated capacity to closely replicate the properties of real blood. Nevertheless, this study utilizes only water.

Representation of the LV simulator is a crucial part of the mock circulation setups [36] shown in Figure 6. The experimental setup utilized Tygon S3 E-3603 NSF-51 by SAINT-GOBAIN (RoadMalvern, PA, USA) tubing featuring an inner diameter of 1 inch, along with an open-air reservoir serving as a substitute for the left atrium.

The Extech laser photo tachometer counter was used to measure the heartbeat. Two distinct types of sensors were utilized in this experiment: a pressure sensor and an ultrasonic flow rate sensor. The ASHCROFT G17M0215F2VAC/30# pressure transmitter (ASHCROFT, Stratford, CT, USA) was utilized for pressure measurements, functioning within a pressure range of −15 psi to 30 psi and a voltage range of 1 to 5V DC. Average fluid flow rates at specified intervals were documented by the flow rate sensors illustrated in Figure 6. The aortic valve was positioned directly after the outlet of the left ventricle simulator, which was powered by a diaphragm pump. To manage fluctuations in atrial pressure throughout the experiments, the left atrium tank was affixed to an adjustable aluminum framework. The data acquisition system used for the experiments was the NI-cDAQ-9178, which included an NI 9215 module from National Instruments Labs Inc. (Austin, TX, USA).

**Figure 7:**
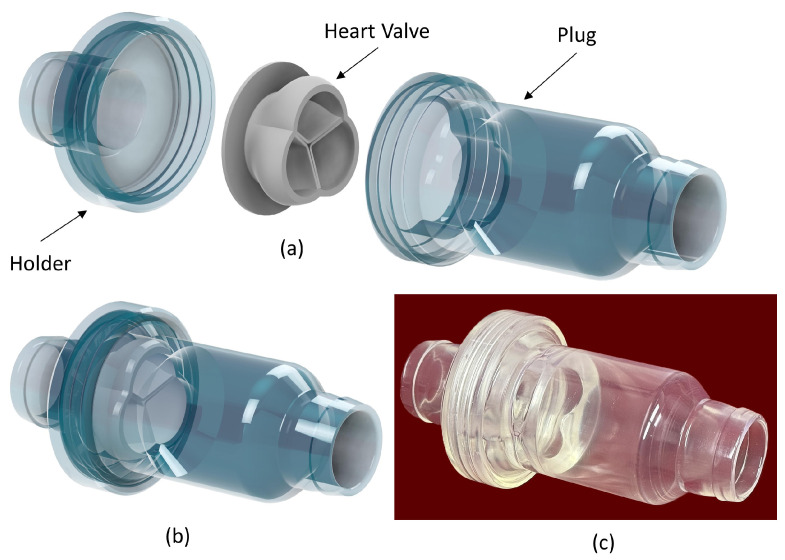
(**a**,**b**) Transparent Heart Valve Chamber Design. (**c**) 3D printed chamber.

The chamber of the heart valve, illustrated in Figure 7, consists of two components: the plug (refer to Figure 7a), which exerts pressure on the heart valve to prevent its movement, and the heart valve holder, which maintains the heart valve’s proper alignment. The inspection camera was utilized to examine the impact of flow rate on the heart valve’s opening and closing mechanisms. To enhance system performance, an air bleeding valve was incorporated to facilitate the release of any trapped air within the system.

### 2.7. 3D Printing Machine Design Refinement

The preliminary design for the 3D silicone printing machine was created, built, and published by Ertas et al. [37]. This step in the design process bridges the gap between the design concept and the project’s detailed design phases. During this phase of the work, system-level and, where possible, component-level design requirements were established and refined. Numerous design improvements were implemented and evaluated to ensure the machine’s functional performance. Specifications comprise all of the component’s needs, such as operational parameters, external dimensions, and controlled interfaces. The machine’s functionality was improved through development and refinement experiments. Analysis of the following variables is part of the refinements for the silicone 3D printer:

(a) Flow rate: Issues related to irregular or insufficient flow rates were examined. Since silicone is typically supplied in 10-ounce cylindrical cartridges that require pressure to be applied to move the silicone, we added an auxiliary pump. This pump uses a NEMA 17 non-captive linear actuator by STEPPERONLINE (New York, NY, USA) with a lead screw that moves through the body of the stepper motor to precisely pressurize the silicone cartridge.
(b) Curing: Challenges with uniform curing were encountered. Several strategies were tried in an attempt to address the inconsistent curing issue, such as experimenting with increasing the number of UV LEDs and changing their angle.
(c) Nozzle Size: To determine what was practical and feasible for nozzle sizes of 0.15, 0.2, 0.3, 0.4, and 0.6 mm, a number of tests were carried out. According to the results, 0.4 mm was the most effective because it had less clogging and could fit more precisely sized geometries. Consistent clogging issues with nozzle sizes of 0.15 and 0.3 mm required higher pressure to minimize clogging, which led to less dimensionally accurate geometries. Although there were significantly fewer clogging issues with the largest nozzle size, 0.6 mm, the flow rate was too high to offer finer geometry features than the 0.4 mm nozzle.
(d) Linear Motion: The linear motion of the X and Y axes was investigated, and lead screws were replaced with ball screws to reduce and eliminate banding, which produces more vibration and step skipping owing to increased friction action.
(e) System Power: The original power source was insufficient for the system. The installation of a second Y-axis motor and three UV LEDs caused the UV LEDs to flicker with each stepper motor movement during the printing process. Upgrading from a generic, lower-quality 24 AC/DC power supply to a Mean Well LRS-350-24 AC/DC switching power supply (Mean Well, Fremont, CA, USA), which is of higher quality, was the solution to this issue.

**Figure 8:**
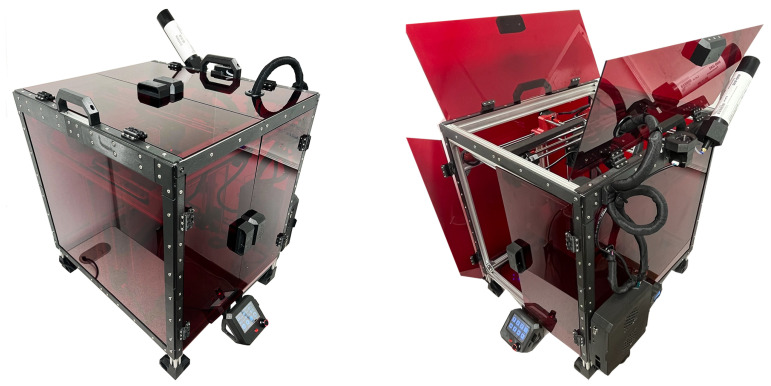
Finished product: a functional 3D silicone printing machine.

Using TD-integrated tools [37], we have successfully designed and built a functional 3D silicone printing machine, as illustrated in Figure 8.

## 3. Results and Discussions

### 3.1. Test Specimen Curing Process

Two methods are employed for the process of curing.

Process #1: This curing process consists of two stages. Initially, silicone test specimens were subjected to pre-curing within a UV resin curing box. Then, the post-curing phase entails the secondary curing of silicone, which is conducted at room temperature ranging from 27 to 31 degrees Celsius for a designated duration. Additionally, the curing area is equipped with four fluorescent lamp units, each containing four UV linear fluorescent tubes activated during the post-curing phase. Two moldings, each housing three test specimens, were produced through 3D printing with high-transparency PETG Plastic, as depicted in Figure 4b. These moldings were then filled with silicone materials, as described earlier. Both sets of molded specimens underwent simultaneous curing for two hours within a UV resin curing box. Following this, the post-curing phase began.

The post-curing process for silicone enhances the physical properties of silicone. Original equipment manufacturers (OEMs) in the healthcare sector emphasize the importance of consistency in their molded products due to regulatory requirements and the inherent risks associated with healthcare applications. The practice of post-curing is widely employed to stabilize silicone and ensure uniformity in the final products [25]. Additionally, post-curing is often utilized within the healthcare industry to prepare samples for biocompatibility assessments. The biocompatibility of silicone elastomers is generally evaluated through extract testing of the pre-cured material. The post-curing process effectively diminishes the volatile concentration of lower molecular weight compounds present in the material [25].

Process #2: This method is known as the UV curing process for silicone, and it takes place in a UV resin curing box for a set amount of time without the requirement for post-curing.

### 3.2. ANOVA Results

After verifying the operational status and performance of the 3D printing machine, we proceeded to conduct tests for ANOVA analysis. A two-level experiment involving three factors was designed and examined to assess the relative effects of print speed, nozzle temperature, and layer height on the print quality of the aortic heart valve. The results of the experimental data collection, derived from the direct observation method, are illustrated in Table 4.

The print speed (P) is detailed in Column 1, where a low setting (1) corresponds to 25 mm/s and a high setting (2) corresponds to 45 mm/s. Column 2 presents the nozzle temperature (N), with a low value (1) of 30 °C and a high value (2) of 120 °C. The layer height (L) is indicated in Column 4, with a minimum of 0.1 mm (1) and a maximum of 0.3 mm (2). A score ($Y_{i}$) of 0 reflects very poor print quality, while a score of 10 signifies very good print quality. A nozzle size of 0.4 mm was utilized for all ANOVA experiments.

**Figure 9:**
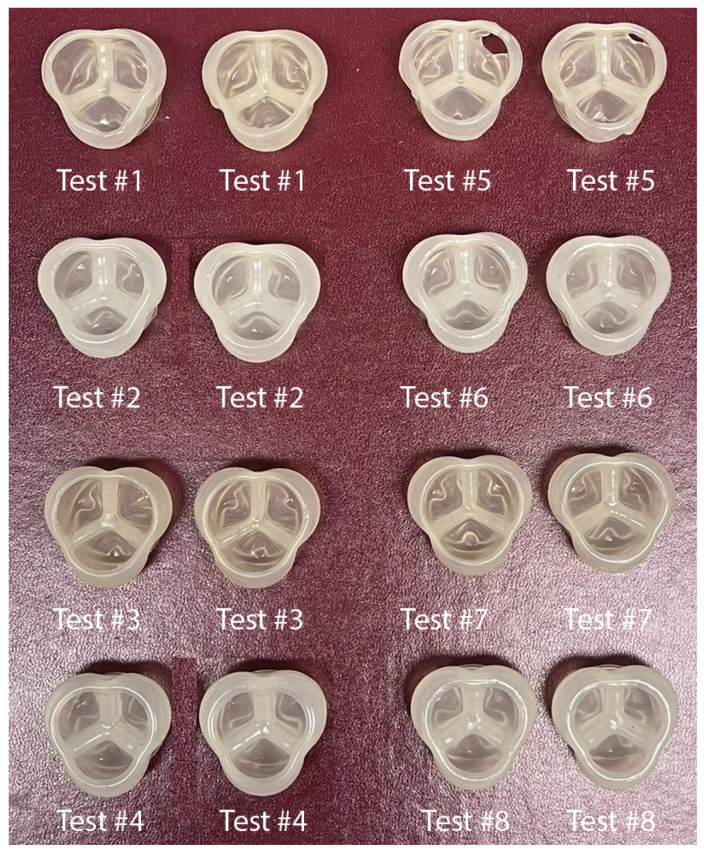
Three-dimensional printed heart valves for ANOVA analysis.

ANOVA Summary for the 3D heart valve printing experiment is shown in Table 5. As illustrated in Figure 9, each ANOVA test was conducted twice to confirm consistency in results. The repeatability of the test outcomes can be verified by examining test number 5, which features a perforation in both upper right corners of the printed heart valve (refer to Figure 9).

The findings from the ANOVA experiment indicate that the quality of the heart valve print is predominantly influenced by layer height (L), followed by print speed (P) and nozzle temperature (N). The overall statistical error is recorded at 2 percent (see Table 5). It was observed that the third test achieved the highest print quality, rated at 10, characterized by a low print speed, high nozzle temperature, and low layer height with a flow rate of 152 steps per millimeter (see Table 5). Throughout each ANOVA experiment, multiple flow rate tests were conducted until the optimal flow rate was established for each scenario, which proved to be the most time-intensive aspect of the experiment.

When the effect of one parameter relies on another, it is referred to as an interaction effect. As illustrated in Table 5, the three main parameters are interrelated. To assess whether a significant interaction exists between print speed and layer height, we will consider the significance threshold of α = 0.05 (95 percent confidence level). The computed value for the interaction between print speed and layer height is as follows:

$$F_{P\times L}=\frac{MS_{P\times L}}{ME_{E}}=\frac{6.125}{0.125}=49$$

In the F distribution table, the F ratio to search for at a 0.05 level of confidence is [38]

$$F_{0.05}\left(\nu_{1}=1,\nu_{2}=1\right)=161.4$$

The interaction effect between print speed and layer height does not reach significance at the 0.05 level, as the calculated F value of 49 is less than the critical value of 161.4 found in the F distribution table.

As indicated in Table 5, the percentage contribution reveals that the layer height factor has the most substantial impact on print quality, as evidenced by the highest variation recorded in this experiment (49). This finding aligns with the F test ratios, which indicate that the layer height factor is more influential than the other factors affecting print quality, given that F = 169 exceeds the ratios of the other factors.

**Figure 10:**
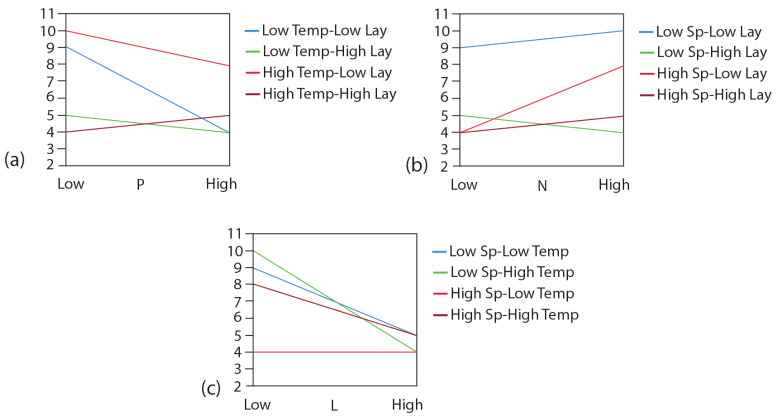
Interaction plot analysis of factors on the quality of heart valve prints.

The distinct impacts of the factors on the quality of heart valve prints are illustrated in the interaction plot analysis presented in Figure 10. The individual effects were assessed by adjusting the value of one factor from low to high while keeping the other two factors at fixed levels. The fixed combinations were defined as High–Low, Low–High, Low–Low, and High–High.

Figure 10a illustrates that the quality of the print diminishes as the print speed increases across three scenarios. Notably, in one scenario characterized by high nozzle temperature and high layer height, there was a marginal improvement in print quality with rising print speed. Except for the combination involving low print speed and high layer height, the quality of the heart valve print showed enhancement with an increase in nozzle temperature (refer to Figure 10b). Furthermore, except for cases involving high print speed and low nozzle temperature, print quality tends to decline as layer height increases (see Figure 10c). In this context, layer height appears to have no significant impact on the print quality of the heart valve.

### 3.3. Tensile Test Results

Two sets of molded silicone test specimens were prepared. After the completion of UV curing for 2 h, one of the two sets of molded silicone test specimens is allowed to cure at room temperature (27–31 °C) for 24 h of post-curing, while the other set undergoes 420 h for post-curing. It is important to note that with an increase in post-curing duration, the specimens tend to become harder and less adhesive. Generally, this results in an increase in material hardness, an enhancement in ultimate tensile strength, and a reduction in elongation. However, the impact of post-curing time on these properties—strength, elongation, and hardness—may vary, potentially leading to either positive or negative effects depending on the specific physical characteristics of the silicone.

**Figure 11:**
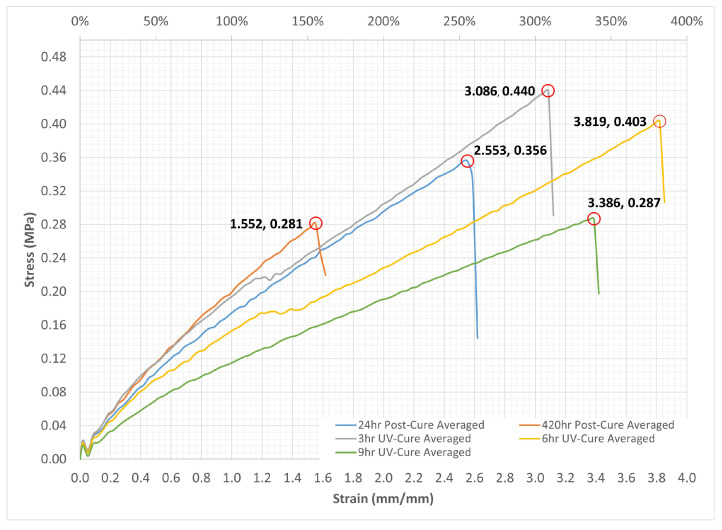
Average stress–strain plots of UV-cured and post-cured silicone.

Figure 11 presents the findings of this study for Process #1, which involves an initial UV cure followed by a post-cure, as well as for Process #2, which consists solely of the UV curing process. This analysis demonstrates the impact of UV curing and the duration of the post-curing period on the mechanical properties of the silicone examined in this study. The figure presents the average stress–strain curves for three silicone samples, comparing those subjected to 24 h and 420 h post-curing with samples that experienced only 3 h, 6 h, and 9 h UV curing.

The results of the Standard Deviation (Std) in conjunction with the averaged readings (Avr) are presented in Table 6. As shown in Table 6, the minimum achievable value for the standard deviation is 0 at 420 h post-cure for hardness. This occurs only when every value within the dataset is identical, leading to no deviation. The other standard deviations for hardness remain consistently low at 0.289, indicating a significant level of consistency within the dataset. The data points are closely grouped around the mean, suggesting minimal spread or variation within the dataset; essentially, most data values are situated near the average value. Tensile strength data also show similar conclusions. The analysis of the 6 h elongation data used in this study indicates that the standard deviation is significantly less than the mean, suggesting a minimal level of dispersion or variation within the dataset.

**Figure 12:**
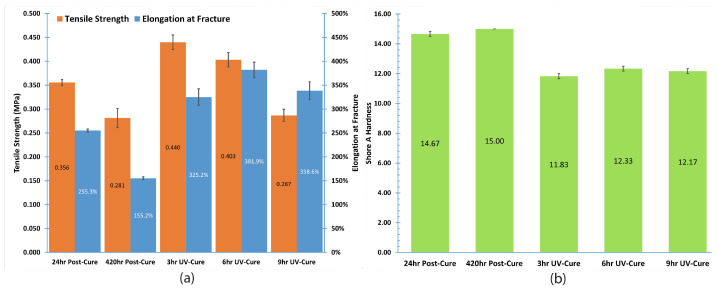
Effect of post-cure and UV-cure on silicone strength, elongation, and hardness.

Figure 12a illustrates a comparison of ultimate tensile strength and elongation for UV-cured silicone material after various curing durations. The data indicate a notable variability in the measured properties, with extended post-cure times leading to a reduction in both strength and elongation. The findings from the 3 h and 6 h UV curing indicate that a longer curing duration leads to a reduction in strength while simultaneously improving the elongation of the silicone material. However, an excessive UV curing time (9 h) results in a decline in these properties. An attempt to utilize an 18 h curing period was made, but the test specimen could not be extracted from the mold due to excessive stickiness. This suggests that an optimal UV curing duration is essential for achieving the best results.

Figure 12b demonstrates that the hardness of the UV-cured silicone material remains relatively stable following each post-cure or UV-cure procedure. Nevertheless, as illustrated in Figure 12b, UV curing leads to a decrease in the material’s hardness compared to post-curing, yielding a softer material that is more suitable for producing heart valves [39].

The error bars, derived from the standard error, are shown to convey the uncertainty associated with the data in relation to each respective bar. Figure 12b demonstrates that the hardness has smaller error bars than those for ultimate tensile strength and elongation in UV-cured silicone material. This indicates that the hardness data have higher precision.

Materials characterized by softness and stretchability at body temperature, along with low glass transition temperatures, high elasticity, and reduced stiffness, are selected to replicate the mechanical properties of the natural heart valve [39]. This paper’s analysis demonstrated that a 6 h UV curing process is more effective for the production of heart valves, leading to increased strength, better elongation, and a more pliable material.

### 3.4. Assessment of 3D Printed Polymeric Heart Valve Performance

**Figure 13:**
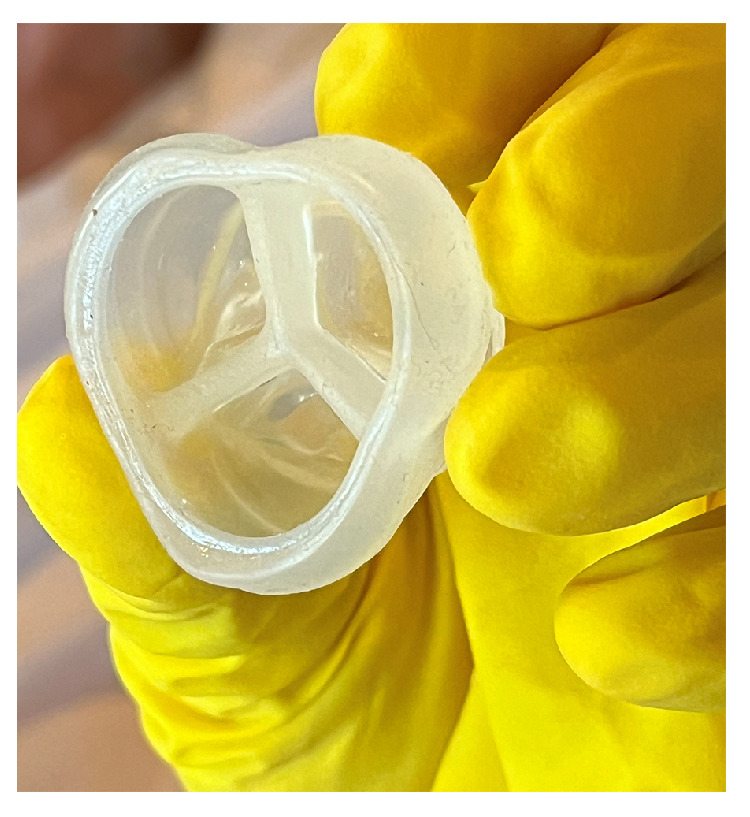
Three-dimensional UV-cured silicone printed aortic heart valve.

The creation of the heart valve illustrated in Figure 13 was based on the results obtained from prior design evaluations. These evaluations encompassed the development of heart valve geometry, ANOVA analysis (test number 3), and the outcomes of a 6 h UV curing process.

The results of ANOVA test number 3, which focused on precise changes in low print speed, high nozzle temperature, and low layer height, were utilized to evaluate the performance of the printed heart valve measuring 23 mm in diameter (see Figure 9).

**Figure 14:**
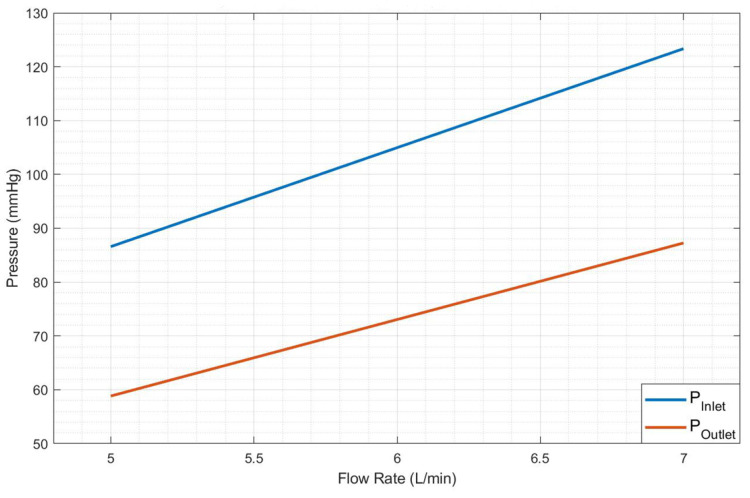
Pressure vs. 0.8 mm leaflet heart valve flow rate.

**Figure 15:**
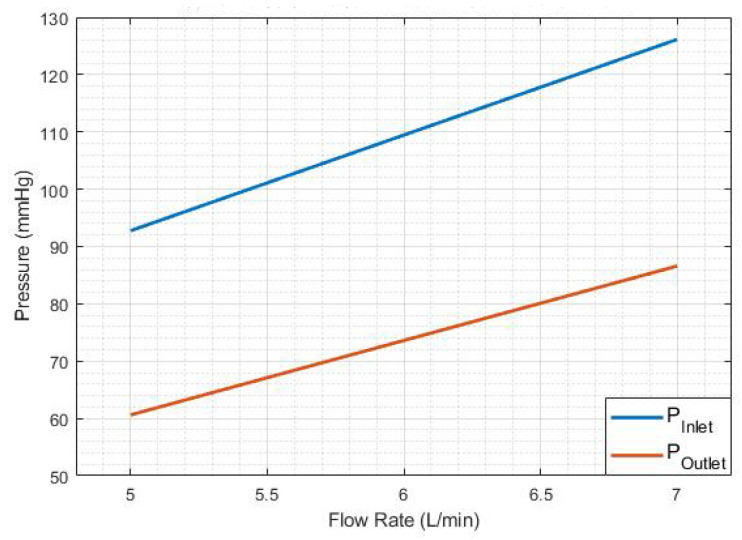
Pressure vs. 1.6 mm leaflet heart valve flow rate.

Polymeric heart valves are a potentially less expensive alternative to mechanical and bioprosthetic ones. For many years, research in prosthetic heart valves has focused on materials with good durability and biocompatibility, with leaflet thickness being a crucial design consideration [40]. In this research, as shown in Figure 13, two different leaflet thicknesses (LT) of the heart valve were examined to model calcium accumulation: 0.8 mm and 1.6 mm. Experimental results are shown in Table 7, Figure 14 and Figure 15.

Table 7 illustrates the recorded data for cardiac cycles (RPM), pressure measurements were taken before and after the heart valve, as well as flow rate information corresponding to each thickness. Figure 14 and Figure 15 illustrate the linear correlation between flow rate and pressure, utilizing the data presented in Table 7.

Calcium may build up on the leaflets of the heart valves, leading to thickening or stiffening of these structures. This accumulation can result in reduced blood flow and may lead to a condition known as stenosis. Varying leaflet thickness changes the bending rigidity, which affects valve functionality. Extremely low stiffness promotes leaflet flapping, which may impair valve performance. Conversely, excessive stiffness makes it difficult to open and close the valve, resulting in increased resistance and lower flow [41].

**Figure 16:**
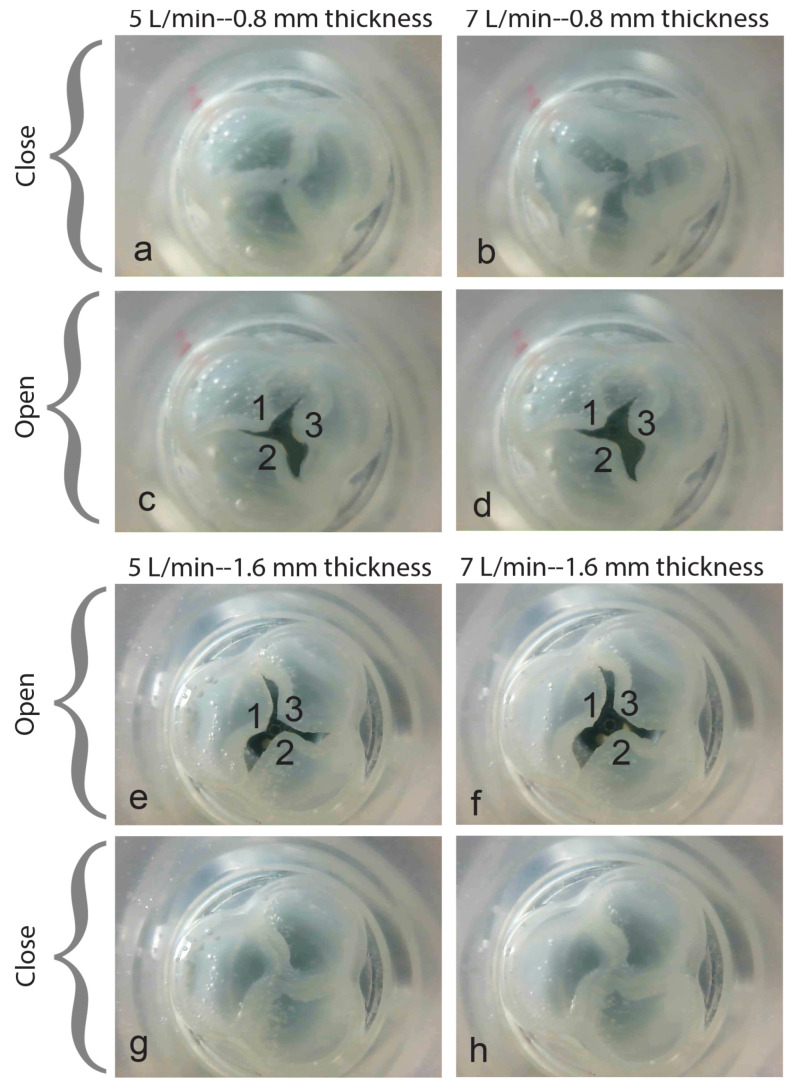
Experimental results of polymeric heart valve openings for different leaflet thicknesses.

This scenario becomes apparent when examining Figure 16e,f, where the presence of thicker leaflets leads to a smaller valve opening in comparison to the thinner leaflets depicted in Figure 16c,d, consequently resulting in a reduction in blood flow. It is important to note that all the valves achieved complete closure without any leakage (see Figure 16a,b,g,h).

To better understand the valve’s functionality, the geometric orifice area (GOA) of the aortic valve opening is evaluated. This evaluation entails projecting the valve’s opening area onto a plane that is perpendicular to the aorta’s axis. However, measuring the valve opening about various parameters, including flow rate and leaflet thickness, proved to be challenging in this research study. Nevertheless, the heart valve functioned properly, achieving desired flow rates of 5 L/min and 7 L/min.

It is important to emphasize that when comparing the valve opening areas, one should focus on the distance between the two leaflets’ tips rather than the total area of the valve opening shown by the dark area in the figures. The shorter the distance between the leaflet’s tips, the smaller the valve opening area will be. For instance, in Figure 16f, the distance between the two leaflet tips (1–3) is shorter than the leaflet’s tips (1–3) in Figure 16d. This suggests that the opening of valve leaflets 1 and 3 in Figure 16d is greater than that of leaflets 1 and 3 in Figure 16f. Upon reviewing Figure 16e,f, it is evident that leaflets 1 and 3 in Figure 16e are not opening sufficiently when compared to leaflets 1 and 3 in Figure 16f. In contrast, Figure 16d demonstrates that all leaflets are effectively participating in the valve opening process. Observations made during the experiment indicated that while the heart valve did not open to its full capacity, all three leaflets were operational and, in fact, able to open to a certain extent.

### 3.5. Heart Valve Inlet and Outlet Pressures

**Figure 17:**
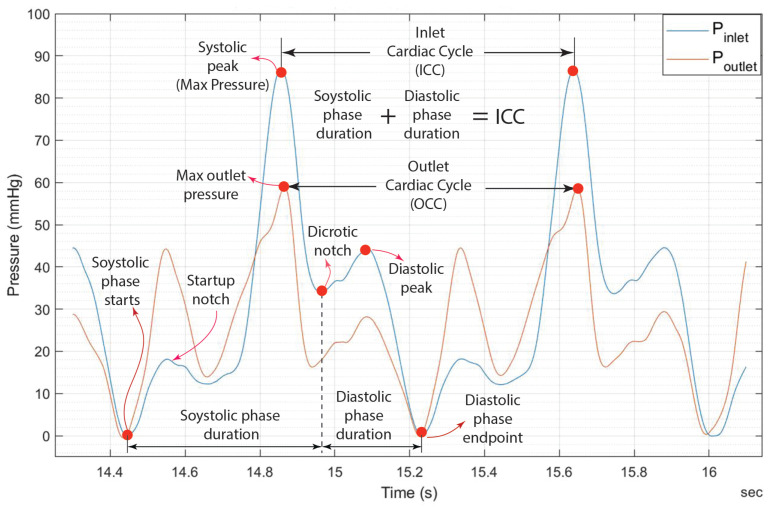
The 0.8 mm thick leaflet heart valve inlet and outlet pressures for 5.0 L/min.

**Figure 18:**
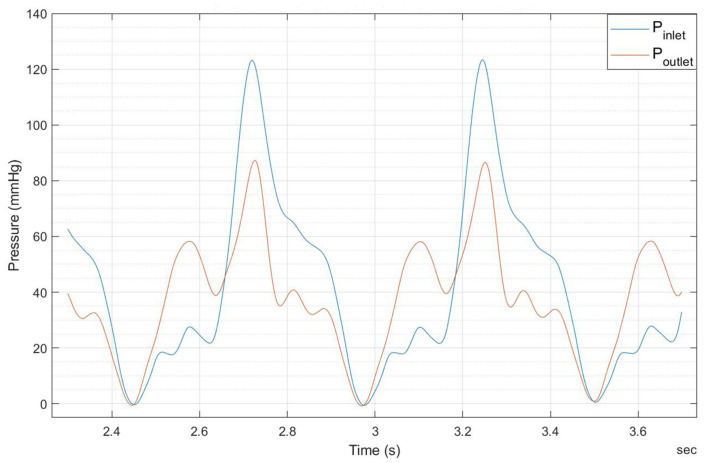
The 0.8 mm thick leaflet heart valve inlet and outlet pressures for 7.0 L/min.

**Figure 19:**
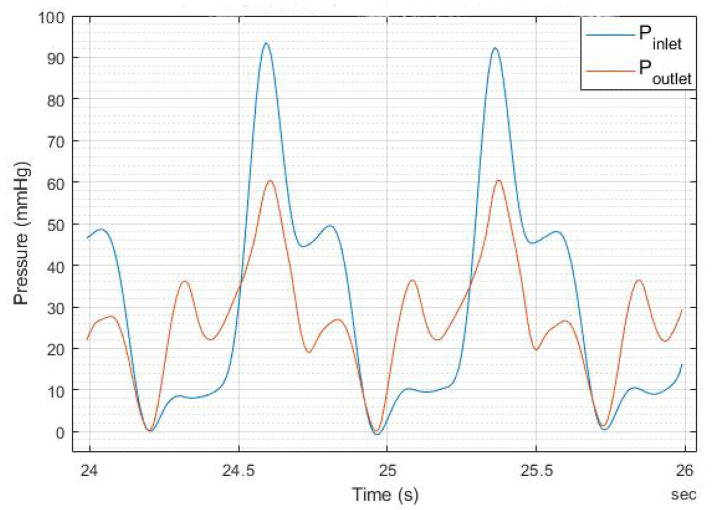
The 1.6 mm thick leaflet heart valve inlet and outlet pressures for 5.0 L/min.

**Figure 20:**
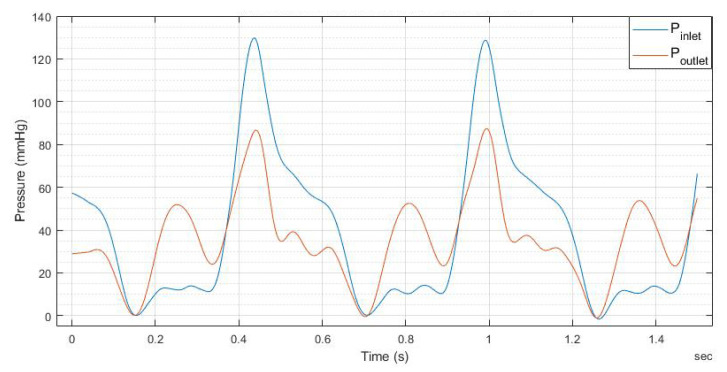
The 1.6 mm thick leaflet heart valve inlet and outlet pressures for 7.0 L/min.

The experimental findings regarding the pressure plots at the inlet and outlet of the heart valve are presented in Figure 17, Figure 18, Figure 19 and Figure 20.

The interval between one heartbeat and the start of the subsequent one is known as the cardiac cycle. This cycle consists of two distinct phases:

1. The phase of ventricular contraction, known as systole;
2. The phase of ventricular relaxation, termed diastole.

The maximum pressure in the arterial system referred to as systolic pressure, is attained during the contraction phase of the cardiac cycle, known as systole, as illustrated in Figure 17. Following the completion of systole, the ventricles relax, leading to a rapid decline in ventricular pressure. The “dicrotic notch” on a heart valve pressure graph shown in Figure 17 signifies a temporary decrease in pressure after the systole, indicating the aortic valve’s closure. The term “diastolic peak” denotes the maximum pressure attained during the relaxation phase of the cardiac cycle. This peak generally occurs shortly after the closure of the aortic valve and before a notable decrease in pressure, which leads to the lowest point observed during diastole (diastolic phase endpoint).

A notch is observed immediately following the onset of systolic pressure at the valve inlet. This occurrence can be attributed to the motor initially consuming a high current during startup, resulting in a small amplitude notch. Subsequently, the plot will stabilize as the systolic pressure rises until it reaches its peak.

Note that Figure 19 and Figure 20 indicate that doubling the thickness of the leaflet mitigates the energy generated during the motor’s initial startup, thereby reducing the notch effect–The maximum notch pressure for the valve inlet for a flow rate of 5 L/min, as illustrated in Figure 17, is approximately 18 mmHg, while the maximum notch pressure for the inlet valve decreases to below 10 mmHg (see Figure 19). A comparable effect of leaflet thickness on the maximum initial notch pressure amplitude is observed in Figure 18 and Figure 20. At a flow rate of 7 L/min, the maximum inlet notch pressure decreases from 28 mmHg, as illustrated in Figure 18, to roughly 12 mmHg (see Figure 20).

As depicted in Figure 17, the startup notch at the valve inlet serves to amplify the amplitude of the notch present at the valve outlet. This effect arises from the considerable resistance encountered when attempting to open the valve at the inlet, in contrast to the significantly lower resistance at the outlet, which permits a greater notch amplitude.

The outlet pressure graphs of the heart valve exhibit a similar trend to those of the inlet pressure, although at a reduced pressure level. The reduction in pressure observed at the heart valve’s outlet is considerable, attributable to the properties of the UV-cured silicone material. In a healthy heart valve, the typical pressure drop at the outlet is generally minimal, approximately ten millimeters of mercury (mmHg). This phenomenon is linked to the heart valve material (UV-cured silicone) employed in this research study. However, it is important to note that the material characteristics of the heart valve utilized in this study do not precisely reflect those of a natural heart valve.

## 4. Conclusions

Major disadvantages of current mechanical and biological heart valve replacements highlight the need for alternative solutions. This study aimed to design, develop, and evaluate a 3D printer capable of utilizing UV-cured silicone for the production of aortic heart valves.

By employing TD-integrated tools, a fully functional 3D silicone printing machine (SPM) was designed and built. The Taguchi–ANOVA approach was used to perform extensive experiments focused on identifying the optimal tuning parameters, namely print speed, nozzle temperature, and layer height, for 3D SPM. The ANOVA experiment’s findings demonstrated that layer height is the most significant factor influencing the quality of the heart valve print. An evaluation of the 3D machine was conducted using two different leaflet thicknesses to assess its dimensional precision. The findings indicated that the machine is operating efficiently.

The process of curing silicone was carried out and assessed to verify its capacity to achieve optimal results. The analysis revealed that a 6 h UV curing period is more effective for the production of heart valves, which leads to increased strength, better elongation, and more pliable material.

The performance of the heart valve was evaluated to ascertain its capability to reach the target flow rates of 5 L per minute and 7 L per minute. The heart valve operated effectively, successfully attaining the specified flow rates.

This research study is certainly not conclusive. The issues of material compatibility, durability, and strength for long life are critical factors that necessitate additional investigation. The suggested 3D printing manufacturing technology utilizing UV-cured silicone presents a distinctive opportunity to produce biomedical implants in the future.

## Figures and Tables

**Table 1:** UV cured silicone specifications.

Description	Appearance	Viscosity	Specific	Tensile Strength	Elongation	Shore A
			Gravity	ASTM D412	ASTM D412	ASTM D2240
		Pa-sec		MPa	%	
UV Cure	Hazy	2.5–6.0	0.950–1.050	0.207–0.621	100–400	15–25
	Viscous Fluid					

Source: https://www.novagard.com/wp-content/uploads/2020/11/TDS-Novagard-800-Series-800-245-UV-Cure-Silicone-v1.6.pdf, accessed on 24 April 2024.

**Table 2:** Three factor $L_{8}$ orthogonal array.

	P	N	P × N	L	P × L	N × L	Error	
Col.→	1	2	3	4	5	6	7	$Y_{i}$
**No.**↓								
1	1	1	1	1	1	1	1	$Y_{1}$
2	1	1	1	2	2	2	2	$Y_{2}$
3	1	2	2	1	1	2	2	$Y_{3}$
4	1	2	2	2	2	1	1	$Y_{4}$
5	2	1	2	1	2	1	2	$Y_{5}$
6	2	1	2	2	1	2	1	$Y_{6}$
7	2	2	1	1	2	2	1	$Y_{7}$
8	2	2	1	2	1	1	2	$Y_{8}$

**Table 3:** Lower and higher limits of main factors.

	Print	Nozzle	Layer
Factors→	Speed (P)	Temperature (N)	Hight (L)
Levels↓	mm/s	°C	mm
Low (1)	25	30	0.1
High (2)	45	120	0.3

**Table 4:** $L_{8}$ orthogonal array for heart valve experiment.

	P	N	P × N	L	P × L	N × L	Error		Flowrate
Col.→	1	2	3	4	5	6	7	$Y_{i}$	(Step/mm)
**No.**↓									
1	1	1	1	1	1	1	1	9	152
2	1	1	1	2	2	2	2	5	220
3	1	2	2	1	1	2	2	10	152
4	1	2	2	2	2	1	1	4	236
5	2	1	2	1	2	1	2	4	144
6	2	1	2	2	1	2	1	4	228
7	2	2	1	1	2	2	1	8	165
8	2	2	1	2	1	1	2	5	262

**Table 5:** ANOVA Summary for 3D heart valve printing experiment.

		Sum of	Mean		
	DOF	Squares	Squares		Percent of
Source	(ν)	$\left({\mathrm{SS}}_{i}\right)$	$\left({\mathrm{MS}}_{i}\right)$	F Test	Variance
P	1	6.125	6.125	49	14
N	1	3.125	3.125	25	7
P × N	1	3.125	3.125	25	7
L	1	21.125	21.125	169	49
P × L	1	6.125	6.125	49	14
N × L	1	3.125	3.125	25	7
Error	1	0.125	0.125		2

**Table 6:** Standard deviation with the averaged results.

DATASET	Shore A Hardness		Tensile Strength (MPa)		Elongation (%)	
	Avg	Std	Avg	Std	Avr	Std
24 h Post-Cure	14.67	0.289	0.356	0.011	255.3%	5.09%
420 h Post-Cure	15.00	0.000	0.281	0.034	155.2%	5.10%
3 h UV-Cure	11.83	0.289	0.440	0.026	325.2%	29.6%
6 h UV-Cure	12.33	0.289	0.403	0.026	381.9%	28.3%
9 h UV-Cure	12.17	0.289	0.287	0.022	338.6%	32.1%

**Table 7:** Experimental results involving a 23 mm aortic heart valve.

Flowrate	Heartbeat	Inlet Pressure	Exit Pressure
(L/min)	(RPM)	(mmHg)	(mmHg)
(LT = 0.8 mm) 5.0	75	86.5874	58.8349
(LT = 1.6 mm) 5.0	72	92.7598	60.6161
(LT = 0.8 mm) 7.0	113	123.3549	87.2601
(LT = 1.6 mm) 7.0	108	126.1436	86.5931

## Data Availability

The data will be made available by the corresponding author on request.
